# Maintenance of Chronic Fatigue Syndrome (CFS) in Young CFS Patients Is Associated with the 5-HTTLPR and SNP rs25531 A > G Genotype

**DOI:** 10.1371/journal.pone.0140883

**Published:** 2015-10-16

**Authors:** Benedicte Meyer, Chinh Bkrong Thuy Nguyen, Aurora Moen, Even Fagermoen, Dag Sulheim, Hilde Nilsen, Vegard Bruun Wyller, Johannes Gjerstad

**Affiliations:** 1 Dept. of Paediatrics, Akershus University Hospital, Oslo, Norway; 2 Dept. of Physical Medicine and Rehabilitation, Oslo University Hospital, Oslo, Norway; 3 Dept. of Anaesthesiology and Critical Care, Oslo University Hospital, Oslo, Norway; 4 Dept. of Paediatrics, Lillehammer County Hospital, Lillehammer, Norway; 5 Dept. of Paediatrics, Oslo University Hospital, Oslo, Norway; 6 Institute of Clinical Medicine, Medical Faculty, University of Oslo, Oslo, Norway; 7 National Institute of Occupational Health, Oslo, Norway; 8 Section for Clinical Molecular Biology, Akershus University Hospital, Oslo, Norway; 9 Department of Biosciences, University of Oslo, Oslo, Norway; University of Marburg, GERMANY

## Abstract

Earlier studies have shown that genetic variability in the *SLC6A4* gene encoding the serotonin transporter (5-HTT) may be important for the re-uptake of serotonin (5-HT) in the central nervous system. In the present study we investigated how the 5-HTT genotype i.e. the short (S) versus long (L) 5-HTTLPR allele and the SNP rs25531 A > G affect the physical and psychosocial functioning in patients with chronic fatigue syndrome (CFS). All 120 patients were recruited from The Department of Paediatrics at Oslo University Hospital, Norway, a national referral center for young CFS patients (12–18 years). Main outcomes were number of steps per day obtained by an accelerometer and disability scored by the Functional Disability Inventory (FDI). Patients with the 5-HTT SS or SL_G_ genotype had a significantly lower number of steps per day than patients with the 5-HTT L_A_L_G_, SL_A_ or L_A_L_A_ genotype. Patients with the 5-HTT SS or SL_G_ genotype also had a significantly higher FDI score than patients with the 5-HTT L_A_L_G_, SL_A_ or L_A_L_A_ genotype. Thus, CFS patients with the 5-HTT SS or SL_G_ genotype had worse 30 weeks outcome than CFS patients with the 5-HTT L_A_L_G_, SL_A_ or L_A_L_A_ genotype. The present study suggests that the 5-HTT genotype may be a factor that contributes to maintenance of CFS.

## Introduction

Chronic fatigue syndrome (CFS) is a long lasting debilitating disorder, characterized by symptoms like disabling fatigue, cognitive impairments, pain and orthostatic intolerance [1]. The diagnosis is based on clinical criteria, and exclusion of other somatic disorder and psychiatric diagnosis. In adolescents, CFS is associated with reduced physical and psychosocial functioning and may be an underlying cause of school drop-out and later workplace absenteeism. Among individuals between 8–17 years the lifetime prevalence of CFS is about 1% [2].

Earlier studies suggest that CFS may be associated with genetic variability in genes important for serotonin (5-HT) signaling [3,4]. Therefore, genetic variability that influences the function of the 5-HT transporter (5-HTT) in the brain might be of importance. Several genetic variants affect the 5-HTT expression that controls the 5-HT re-uptake. One of these is the 5-HTT promoter repeated-length polymorphic region (LPR) [5]. This repeated-length region consists of 22 base pairs (bp). Two common allelic variants have been described, a short (S) allele of 14 repeats and a long (L) allele of 16 repeats [6]. The S allele seems to reduce the transcription rate and lower expression of the transporter in cell membranes [7,8].

In the gene encoding the 5-HTT there is also a single nucleotide polymorphism (SNP), an A to G substitution rs25531 in the promoter [5]. This A to G shift creates a possible binding site for a transcription factor, activating enhancer-binding protein 2 (AP2) [5]. AP2 is a transcription inhibitor found to bind to the sequence if the G substitution is present [9]. Therefore both the S allele of 14 repeats, and the SNP that introduces the A to G substitution rs25531 (_G_), may reduce the transcription rate of the 5-HTT gene *SLC6A4* in neuronal tissues. The G allele has, however, a low frequency, and seems to be present only on the L alleles of 16 repeats.

Earlier studies have suggested that negative psychosocial stressors may have a more pronounced impact on individuals with the 5-HTT SS and SL_G_ genotype than other individuals [10]. Moreover, variation in the 5-HTT gene *SLC6A4*, indicating decreased transcription, may modulate anxiety and negative affect [11,12]. However, individuals with the low 5-HTT transcription genotype also seem to have a more pronounced effect of positive environmental factors. Therefore, it seems likely that the low 5-HTT transcription genotype is associated with increased neuroplasticity making individuals more susceptible to environmental influences [13].

Difficulty with performance of daily activities (in home, school and social domains) may be associated with anxiety. Based on the link between anxiety and the 5-HTT SS and SL_G_ genotype [12], we hypothesized that CFS patients with this genotype, would have a more pronounced reduction in the daily activities than other CFS patients. Hence, daily activity and disability were defined as main outcomes in the present study. The aim of the present study was to examine how the 5-HTTLPR and SNP rs25531 A > G genotype influences physical and psychosocial functioning in CFS patients.

## Materials and Methods

### Subjects

The present study was a part of the NorCAPITAL-project (The Norwegian Study of Chronic Fatigue Syndrome in Adolescents: Pathophysiology and Intervention Trial). As previously described [14], all patients were recruited from The Department of Paediatrics at Oslo University Hospital, Norway, a national referral center for young CFS patients (12–18 years). The intervention used in the trial was orally administered clonidine (25 μg or 50 μg for body weight <35 kg or >35 kg, respectively, twice daily). Since the double-blinded clonidine intervention did not have any effect on the physical and psychosocial functioning in the patients [14], the clonidine treatment was only considered as a potential co-factor. A broad case definition of CFS, requiring three or more months of unexplained, disabling fatigue worsened by physical or mental exertion, was applied [15].

Data were collected in the period March 2010 until October 2012. Written, informed consent was obtained from all the participants and their parents. The project was approved by the Norwegian National Committee for Ethics in Medical Research and the Norwegian Medicines Agency. A total of 176 patients (all Caucasian) with CFS were assessed for eligibility, of which 120 fulfilled the inclusion criteria, started treatment at the hospital and were included in the present study (for further information, see Sulheim et al 2014 [14]). In addition, 38 age and gender balanced healthy controls from the same geographical area served as a reference group.

At inclusion the data set of the 120 patients was 100% complete. However, 14 patients became drop-outs between inclusion and 8 weeks and another 5 patients became drop-outs between 8 weeks and 30 weeks. The total number of drop-outs during the follow-up was therefore 19 patients i.e. 16%. The number of patients at 30 weeks was 101.

### Clinical measures

In the present study we focused on two main outcomes; daily activity and disability. Daily activity were measured by mean number of steps per day (number of steps), obtained by an accelerometer. Such accelerometers provide reliable data among patients with impaired physical capacity [16]. According to present recommendations, a recording period of seven consecutive days was used [17]. The participants were instructed to wear the unit permanently (i.e. also during the night). Moreover, the questionnaire Functional Disability Inventory (FDI) was used to measure disability. The FDI is a 15-item self-report inventory assessing difficulty with the performance of daily activities (in home, school and social domains) such as “doing chores at home,” “being at school all day,” or “walking the length of a football field.” Items were rated on a 5-point Likert scale, ranging from 0 to 4 (“No Trouble” to “Impossible”) and summed to create a total score (range 0–60) with higher scores indicating greater pain-related disability. The FDI score may be considered as a global measure of children’s and adolescent’s physical and psychosocial functioning in everyday social roles [18].

### qPCR

The blood samples (3ml; antecubital venous puncture at 30 weeks, 101 patients), were collected in Tempus Blood RNA Tubes (for further information, see Sulheim et al 2014 [14]). Total RNA was extracted by Tempus spin RNA isolation Reagent kit (4378926, Life Technologies/Applied Biosystems), and (for 92 patients; those with RIN>7) converted into cDNA by High-Capacity cDNA Reverse Transcription Kit (4374966, Life technology). Primers were designed based on Ensembl transcript ENST00000261707 sequence and OligoEvaluator Tools (Sigma Aldrich), and checked for specificity by performing a BLAST search. To avoid amplification of possible genomic DNA contamination, PCR primers were designed to span a boundary region of two continuous exons. The gene expression of the target gene 5-HTT was normalized to the expression of the reference gene GAPDH. The primer sequences were: 5-HTT forward; AACTG CTACC AAGAT GCCCT, 5-HTT reverse; CTCAG CCATG TAACC GAGCA, GAPDH forward; CCAAC TGCTTA GCACC CCT and GAPDH reverse; TGGCA TGGAC TGTGG TCAT. The quantitative PCR analysis was performed on an ABI 7900 HT (Applied Biosystems, Foster City, California, USA), 40 cycles x (95°C 5s, 60°C 15s), using the Evagreen Sso Fast Master mix (Biorad Laboratories, CA-USA); 05μM primer mix; 2μl cDNA in a total reaction volume of 10 μL.

### Genotyping

Collection of saliva and extraction of genomic DNA was done using OrageneDNA sample collection kit (DNA Genotech Inc. Kanata, Ontario, Canada) according to the manufacturer’s instructions. To determine the length of the polymorphic promoter region of the 5-HTT, the DNA sequence was first amplified by polymerase chain reaction (PCR) and then separated by gel electrophoresis. PCR was carried out in a total volume of 25 μl containing ~60 ng of genomic template, 6.25 pmol of each primer and 1x Taq DNA Polymerase Master Mix (VWR international, Dublin, Ireland). The forward primer sequence was 5’–GGCGT TGCCG CTCTG AATGC- 3’ and the reverse primer sequence was 5’–GAGGG ACTGA GCTGG ACAAC CAC- 3’ (DNA technology A/S, Risskov, Denmark). Samples were amplified on a Perkin Elmer GeneAmp PCR 2400 system following an initial denaturing step for 3 min at 95°C. The amplification consisted of 40 cycles including denaturing at 95°C for 40 s, annealing at 60°C for 20 s and elongation at 72°C for 80 s, as previously described in. The described PCR yielded a long (529 bp) and a shorter (486 bp) fragment [19]. After four hours separation at 100 V on a 2.5% agarose gel (MetaPhor Agarose, Lonza cologne GmbH, Cologne, Germany), GelRed dye was added and the fragments were visualized by UV light (Biotium Inc, California, USA). The PCR 100 bp low ladder (Sigma-Aldrich CO, St. Louis, Mo, USA) was used to determine the length of the fragments.

SNP genotyping was carried out using custom TaqMan SNP genotyping assays (Applied Biosystems, Foster City, CA, USA). Approximately 10 ng genomic DNA was amplified in a 5 μl reaction mixture in a 384-well plate containing 1x TaqMan genotyping master mix (Applied Biosystems) and 1x assay mix, the latter containing the respective primers and probes. The probes were labelled with the reporter dye FAM or VIC to distinguish between the two alleles. Approximately 10% of the samples were re-genotyped and the concordance rate was 100% (for more technical details see Olsen et al 2012 [20]).

### Data analysis and statistics

The data are shown by means ± SEM. The 5-HTT mRNA in blood of the patients, the distribution of the 5-HTT genotype among patients versus healthy controls and characteristics of the patients grouped by genotype, were examined by linear regression, Pearson Chi-square and/or Student’s t-test. The main outcomes; i.e. number of steps and FDI measurements over time were compared regarding genotype by repeated measures ANOVA, between-subjects effect. Missing data at first or second follow-up were replaced by individual data from inclusion or previous follow-up, respectively. When sphericity assumption was not met, a Greenhouse-Geisser correction was applied. Separate analyses were performed to check for potential effects of covariates age, gender, Body Mass index (BMI) and treatment (covariates with p≤0.1 were kept in the final model). Fisher’s LSD post hoc tests using Bonferroni correction were applied to analyze the genotype stratified data at each time point. Statistical analyses were performed using SPSS version 21.

## Results

**Fig 1:**
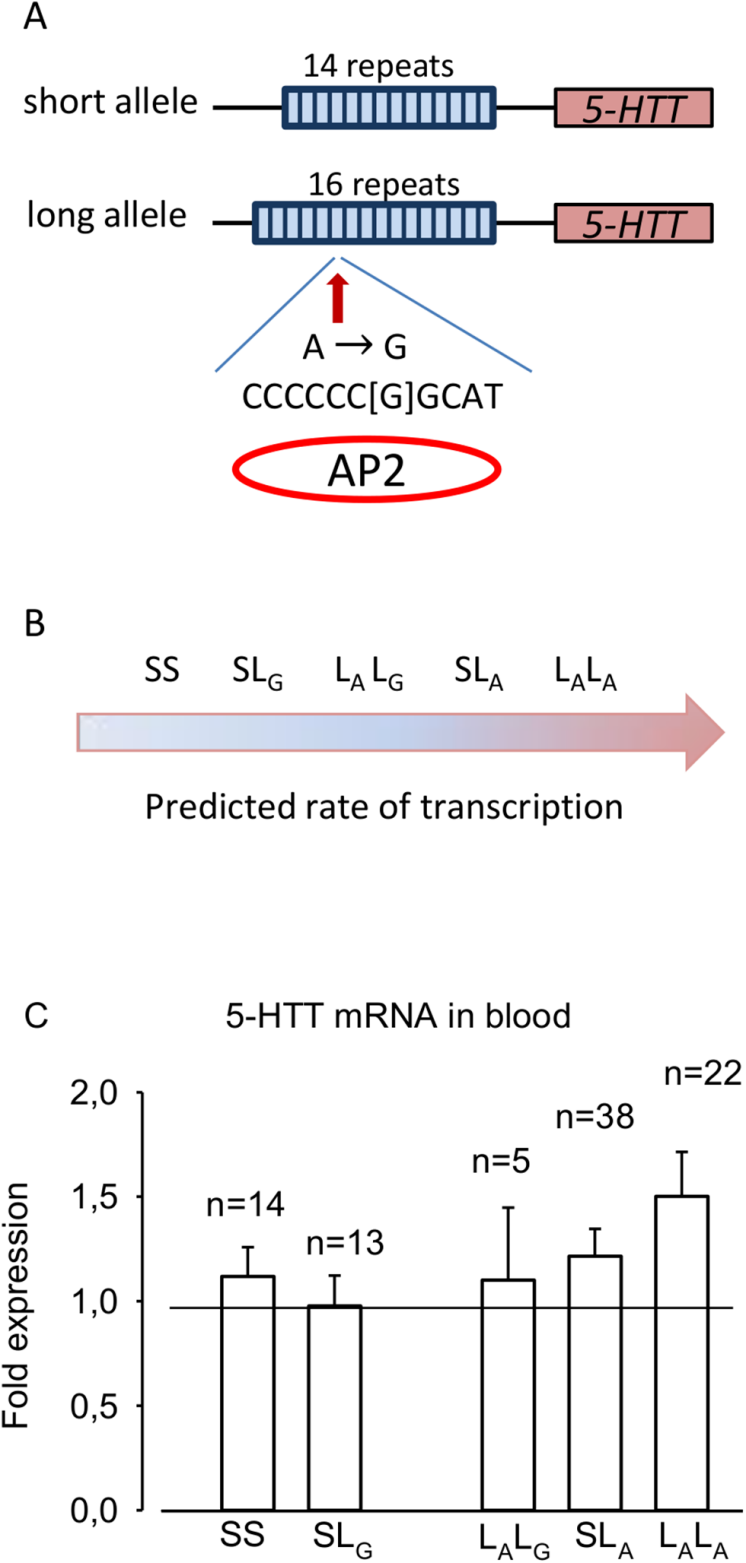
Role of polymorphisms in the 5-HTT promotor region. **A)** The 5-HTT short (S) allele consists of 14 repeats at 22 bp while the long (L) allele consists of 16 repeats and a possible A to G SNP. AP2 is a transcription inhibitor found to bind the sequence if a G substitution is present [5]. **B)** Five possible allele combinations ranged by their predicted rate of transcription. Frequency of the allele combinations in the Caucasian population: SS: 20%, SL_G_: 4%, L_A_L_G_: 5%, SL_A_: 41% and L_A_L_A_: 26% [6]. A: adenosine, G: guanosine, AP2: activating enhancer-binding protein. **C)** 5-HTT mRNA in blood in the CFS patients.

In total 86 females and 34 males with CFS were included in the present study. As expected, the patients were less active than the 38 individuals in the matched control group (patients; 4662 ± 220 number of steps per day and 23 ± 0,84 in FDI score; controls 11293 ± 603 number of steps per day and 1.3 ± 0.46 in FDI score). A weak association between the predicted transcription rates and the 5-HTT mRNA expression in blood in the CFS patients (linear regression; beta = 0.10, p = 0.045) was observed (Fig 1A, 1B and 1C).

**Fig 2:**
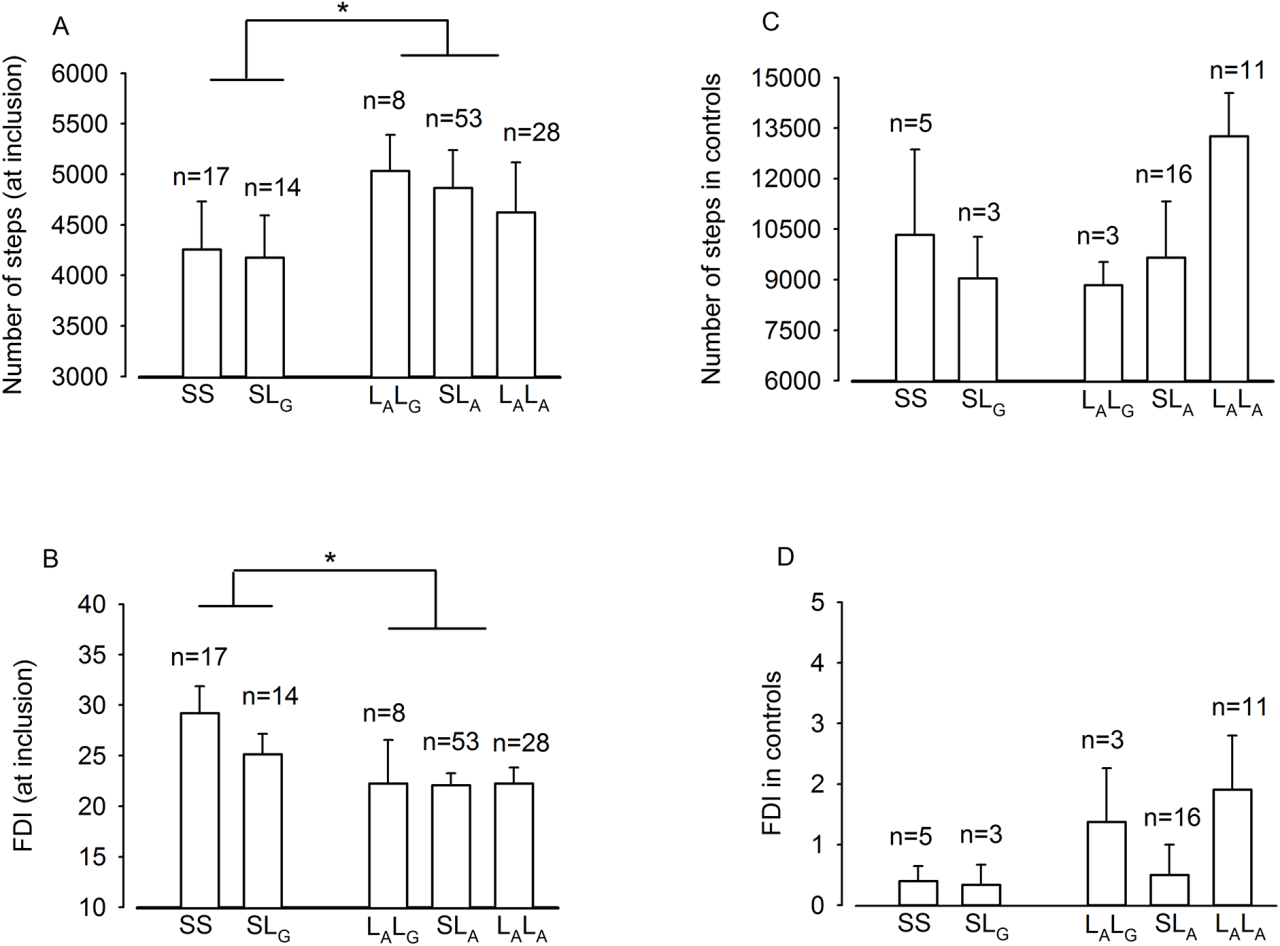
Daily activity and disability scores in 5-HTT genotype stratified data; SS, SL_G_, L_A_L_G_, SL_A_ and L_A_L_A_. **A and B**) Mean number of steps per day and Functional Disability Inventory (FDI) score in the patients at the time of inclusion. **C and D**) Mean number of steps per day and Functional Disability Inventory (FDI) score in the reference group i.e. healthy control subjects. Data are given as means ± SEM (Student’s t-test, SS+SL_G_ versus L_A_L_G_+SL_A_+L_A_L_A_ *p<0.05).

At inclusion the 31 patients with the SS or SL_G_ genotype had a lower number of steps per day and a higher FDI score than the 89 patients with the L_A_L_G_, SL_A_ or L_A_L_A_ genotype (Fig 2A and 2B). No genotype effects were observed in the healthy controls (Fig 2C and 2D). Moreover, the frequency of the SS or SL_G_ genotype was not significantly higher in the patients than in the healthy controls; p = 0.55 (Table 1).

**Table 1:** Patients versus healthy controls; 5-HTT genotype distribution.

	SS/SL_G_	L_A_L_G_/SL_A_/L_A_L_A_	p-value^a^
**Patients (%)**	31(26)	89(74)	0.55
**Controls (%)**	8(21)	30(79)	

^a^ Pearson Chi-square test

Among the potential covariates gender, age, BMI and treatment, gender–the only covariate with p≤0.1 (Table 2)–was included as a covariate in the further analyses. Thus, the over-representation of females with the SS or SL_G_ genotype was corrected for. Notably, 26 of 86 females, but only 5 of 34 males were SS or SL_G_ carriers (Table 3).

**Table 2:** Covariates considered in the analyses. Repeated measures ANOVA.

Outcome measure	Covariates	Within subjects effects. p-values	Between subjects effects. *p*-values	Included in final model. yes/no
**Number of steps**	Age	0.152	0.419	No
	Gender	0.357	0.019	Yes
	BMI	0.650	0.391	No
	Clonidine treatment	0.352	0.120	No
**FDI**	Age	0.071	0.707	No
	Gender	0.037	0.072	Yes
	BMI	0.477	0.615	No
	Clonidine treatment	0.633	0.118	No

The table gives an overview of the association between covariates and the two outcome measures: Number of steps and Functional disability inventory (FDI). Covariates with a value p ≤ 0.1 were included in the final model.

**Table 3:** Characteristics of patients grouped by genotype; 5-HTT SS/SL_G_ versus 5-HTT L_A_L_G_/SL_A_/L_A_L_A_.

	5-HTT SS/SL_G_ n = 31	5-HTT L_A_L_G_/SL_A_/L_A_L_A_ n = 89	*p*-values
**Gender, men/women (%)**	5/26 (0.16/0.84)	29/60 (0.33/0.67)	0.080^a^
**Mean age (min/max)**	15.5 (12.8–17.9)	15.3 (12.0–17.9)	0.521^b^
**Mean BMI (min/max)**	20.7 (16–32.3)	21.2 (14.3–34.1)	0.199^b^
**Clonidine treatment yes/no (%)**	15/16 (0.48/0.52)	45/44 (0.51/0.49)	0.835^a^

^a^Pearson Chi-square test

^b^Unpaired student’s t-test.

**Fig 3:**
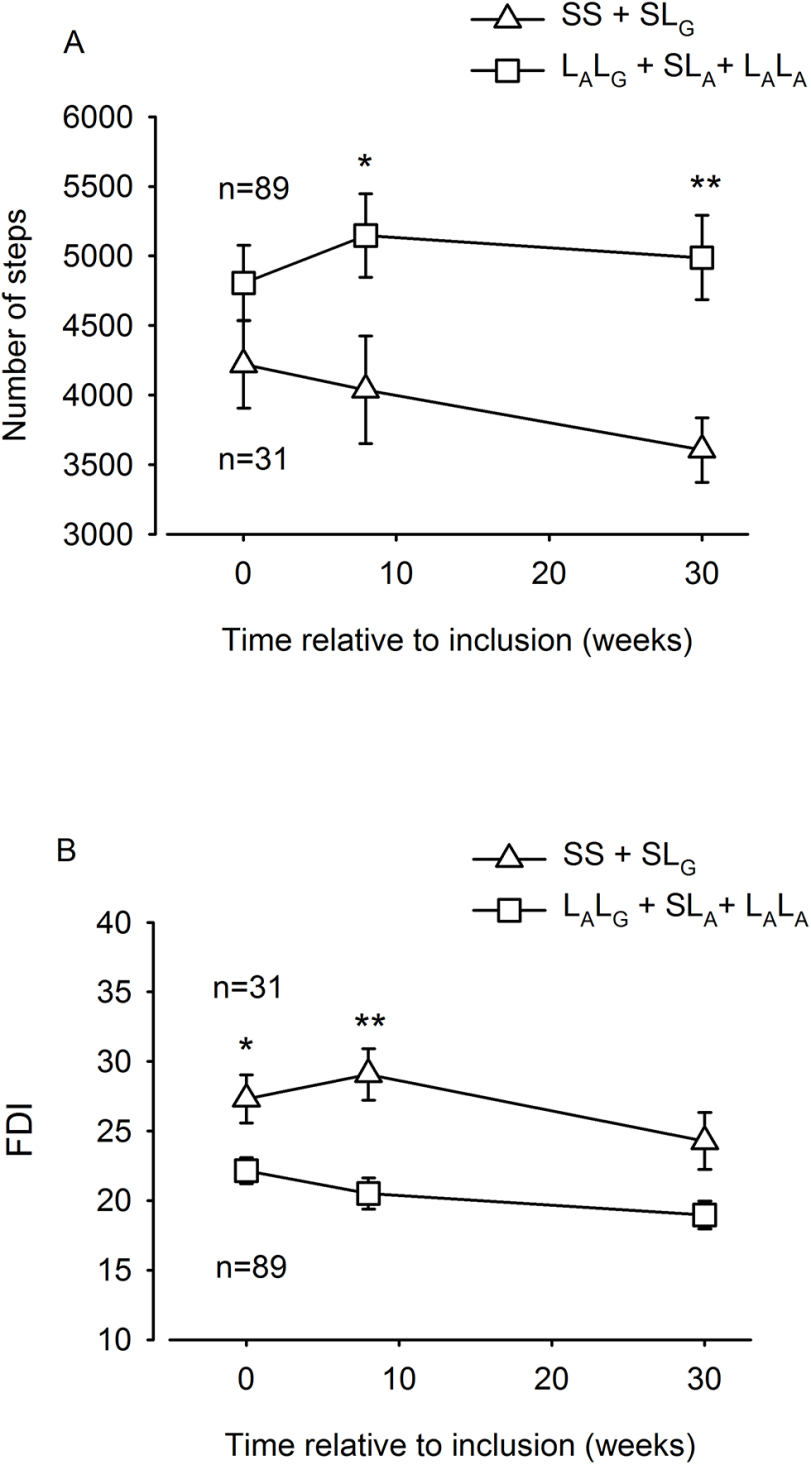
The time course for outcome measures grouped as 5-HTT SS+SL_G_ genotype, and 5-HTT L_A_L_G_+SL_A_+L_A_L_A_ genotype following inclusion. **A**) Number of steps (repeated measure ANOVA, F(1,116) = 7.23, p = 0.008). **B**) Functional disability inventory, FDI (repeated measure ANOVA, F(1,115) = 7.81; p = 0.006). Data are given as means ± SEM (Fisher’s LSD post hoc test using Bonferroni correction *p<0.05; **p<0.01.)

At inclusion, 8 weeks and 30 weeks, a clear difference between the 5-HTT SS or SL_G_ genotype versus the 5-HTT L_A_L_G_, SL_A_ or L_A_L_A_ genotype was observed (Fig 3A and 3B). Patients with the SS or SL_G_ genotype had a significantly lower number of steps per day and also a significantly higher FDI score than patients with the L_A_L_G_, SL_A_ or L_A_L_A_ genotype when gender was taken into account as a covariate (repeated measures ANOVA, between-subjects effect, steps per day F(1,116) = 7.23, p = 0.008; FDI score F(1,115) = 7.81; p = 0.006).

Additional analyses of the data at inclusion, 8 weeks and 30 weeks from only the 86 females, without the 34 males, gave the same results. Female patients with the SS or SL_G_ genotype had a significantly lower number of steps per day and also a significantly higher FDI score than female patients with the L_A_L_G_, SL_A_ or L_A_L_A_ genotype (repeated measures ANOVA, females only, between-subjects effect, steps per day F(1,83) = 9.73; p = 0.002; FDI score F(1,82) = 9.29; p = 0.003).

## Discussion

The purpose of the present study was to examine how the 5-HTTLPR and SNP rs25531 A > G genotype influences CFS. In accordance with previous studies of Caucasians or European Americans indicating a general 5-HTT SS or SL_G_ genotype frequency of about 25% [6,10], the same genotype frequency was 26% in the patients and 21% in the controls. Thus, the 5-HTT genotype cannot explain why some individuals develop CFS. Interestingly however, we found that CFS patients with the 5-HTT SS or SL_G_ genotype had a more pronounced reduction in physical and psychosocial functioning than patients with the 5-HTT L_A_L_G_, SL_A_ or L_A_L_A_ genotype. This suggests that patients with the 5-HTT SS or SL_G_ genotype have a less favorable 30 weeks outcome than patients with the 5-HTT L_A_L_G_, SL_A_ or L_A_L_A_ genotype.

Earlier *in-vitro* studies–as well as the present analyses of 5-HTT expression in blood–suggest that the transcription rate in Caucasians of the 5-HTT gene *SLC6A4* may increase in the following order; SS, SL_G_, L_A_L_G_, SL_A_ and L_A_L_A_ [5]. Moreover, earlier *in-vivo* positron emission tomography studies have provided evidence that the SS and SL_G_ subjects may display decreased expression of 5-HTT in the midbrain [21]. The result is a reduced 5-HT re-uptake, and a higher concentration of extracellular serotonin. This might have two possible consequences: 1) either increased negative feedback as a result of more available 5-HT near the presynaptic autoreceptors, or 2) increased signaling because of a higher concentration of 5-HT near the postsynaptic receptors of the cell membrane.

Depending on the localization of the 5-HTT relative to the 5-HT autoreceptors, this might decrease or increase the 5-HT signaling in the CNS. Hence, genetic variability in the gene encoding the 5-HTT has consequences that may be associated with mental health. Previous data have demonstrated that the S allele of the 5-HTTLPR may be linked to depression [22,23] and suicidality in relation to stressful life events [22]. Earlier studies also suggest that the S allele of the 5-HTTLPR is associated with neurocognitive impairment [24] and bipolar disorders [25].

In addition, variation in the 5-HTT gene *SLC6A4* may modulate attention to threat [26], anxiety, negative affect and fear [10–12]. Moreover, the SS and SL_G_ genotype seems to be associated with higher neuroticism [11], and a more serious symptomatic profile in patients who suffers from posttraumatic stress disorders [10]. Ultimately, it has also been suggested that the 5-HTT SS and SL_G_ genotype may be associated with increased neuroplasticity making individuals more susceptible to environmental influences [13]. Hence, psychosocial aspects, bullying and stressful life events may have a more pronounced impact on adolescents with the 5-HTT SS and SL_G_ genotype than other individuals. These data, together with the observations of the present study, indicate that the 5-HTT SS and SL_G_ genotype may be a factor that contributes to the maintenance of CFS.

Genetic variability that affect the re-uptake of 5-HT may also be important for the regulation of the hypothalamic-pituitary-adrenal (HPA) axis [27], which in turn can affect the symptom burden [28]. Earlier findings show that young CFS patients seem to have an increased efferent sympathetic nerve activity that stimulate the adrenal medulla and increase the secretion of noradrenaline/adrenaline [29]. Moreover, previous data have demonstrated that CFS patients often have an attenuation of the HPA-axis, which involves a reduced release of both ACTH and cortisol during psychosocial stress [30]. In addition, CFS patients may have changes in the hormonal systems controlling blood volume and osmolality [31]. This supports the theory that functional changes in the brainstem may be a part of the etiology in CFS.

An increasing number of studies suggest that changes in the central nervous system are important for CFS, including abnormalities in the serotonergic system and disturbed control of the HPA axis [30]. In line with this hypothesis, our study indicates that individual characteristics of the serotonergic system due to 5-HTTLPR S allele and the SNP rs25231 A > G, important for the expression of the 5-HTT in the midbrain [21], might contribute to maintenance of CFS. However, the frequency of the 5-HTT SS and SL_G_ genotype do not increase the risk of CFS outbreak. Hence we think that the 5-HTT SS and SL_G_ genotype rather may be associated with increased susceptibility to negative environmental influences, inactivity and social isolation.

In conclusion, our data demonstrate that the 5-HTT genotype is associated with daily activity and disability score in young CFS patients. Clearly, CFS patients with the 5-HTT SS or SL_G_ genotype had a worse 30 weeks outcome than CFS patients with the 5-HTT L_A_L_G_, SL_A_ or L_A_L_A_ genotype. The present study suggests that the 5-HTT genotype may be a factor that contributes to maintenance of CFS.
